# A receptor fusion protein for the inhibition of murine oncostatin M

**DOI:** 10.1186/1472-6750-11-3

**Published:** 2011-01-11

**Authors:** Liv Brolund, Andrea Küster, Sabrina Korr, Michael Vogt, Gerhard Müller-Newen

**Affiliations:** 1 Institute of Biochemistry and Molecular Biology, Medical School, RWTH Aachen University, Aachen, Germany

## Abstract

**Background:**

Most cytokines signal through heteromeric receptor complexes consisting of two or more different receptor subunits. Fusion proteins of the extracellular parts of receptor subunits turned out to be promising cytokine inhibitors useful in anti-cytokine therapy and cytokine research.

**Results:**

We constructed receptor fusion proteins (RFP) consisting of the ligand binding domains of the murine oncostatin M (mOSM) receptor subunits mOSMR and mgp130 connected by a flexible linker as potential mOSM inhibitors. mgp130 is a shared cytokine receptor that is also used by other cytokines such as IL-6 and leukemia inhibitory factor (LIF). In this study we compare four types of mOSM-RFPs that contain either domains D1-D3 or domains D2-D3 of mgp130 and are arranged in two ways. Domain D1 of mgp130 turned out to be dispensable for mOSM-binding. However, the arrangement of the two receptor subunits is essential for the inhibitory activity. We found mOSM induced STAT3 phosphorylation to be suppressed only when the mOSMR fragment was fused in front of the mgp130 fragment.

**Conclusions:**

mOSM-RFP consisting of D1-D4 of mOSMR and D2-D3 of mgp130 is a highly potent and specific inhibitor of mOSM. Since mOSM-RFP is encoded by a single gene it offers numerous possibilities for specific cytokine inhibition in gene delivery approaches based on viral vectors, transgenic animals and finally gene therapy.

## Background

Cytokines are central mediators of the immune system. Anti-cytokine therapies are aimed at the specific inhibition of a cytokine that has been identified to be critically involved in the initiation, maintenance or progression of a disease. Most cytokines signal through heteromeric receptors consisting of two different receptor chains. We have developed a new class of cytokine inhibitors based on the fusion of the ligand-binding domains of cytokine receptors by a flexible linker [1]. The prototypic receptor fusion protein (RFP) directed against human interleukin-6 (hIL-6-RFP) turned out to be a highly specific and highly potent inhibitor of hIL-6 [2]. Based on this original approach further RFP have been generated by others for the inhibition of human oncostatin M [3] and most recently human interleukin-31 [4]. In a different but related approach so called cytokine traps have been generated by the fusion of soluble receptors through Fc-fragments [5].

For the validation of the RFP approach in murine animal models *in vivo *RFP directed against murine cytokines are required. RFPs based on human receptor proteins are not useful for this purpose because murine cytokines usually do not bind to the human receptors. Therefore, we concentrated on the generation of receptor fusion proteins for the inhibition of murine cytokines. We described mLIF-RFP [6] for the inhibition of murine leukemia inhibitory factor (mLIF) and recently mIL-6-RFP [7] for the inhibition of murine IL-6 (mIL-6).

Oncostatin M (OSM) is a pro-inflammatory cytokine of the IL-6 family implicated in rheumatoid arthritis [8], lung fibrosis [9] and skin disease [10]. OSM is secreted by activated T-cells [11], macrophages [12], neutrophils [13] and synovial fibroblasts from patients with rheumatoid arthritis [14]. The murine OSM receptor consists of two receptor proteins [15], the OSM-specific OSMR and gp130, the common signalling receptor subunit of the IL-6 family of cytokines. OSM signals through the Jak/STAT pathway resulting in the activation of STAT3 and STAT5. ERK1/2 and p38 MAP kinases are also activated in response to OSM [16].

Here we describe the generation of a novel inhibitor for murine OSM, mOSM-RFP, that is based on the fusion of murine OSMR and murine gp130 fragments. mOSM-RFP will be a useful tool for the investigation of the role of OSM in murine models of human diseases.

## Results

### 1. Design and expression of murine oncostatin M receptor fusion proteins (mOSM-RFPs)

**Figure 1:**
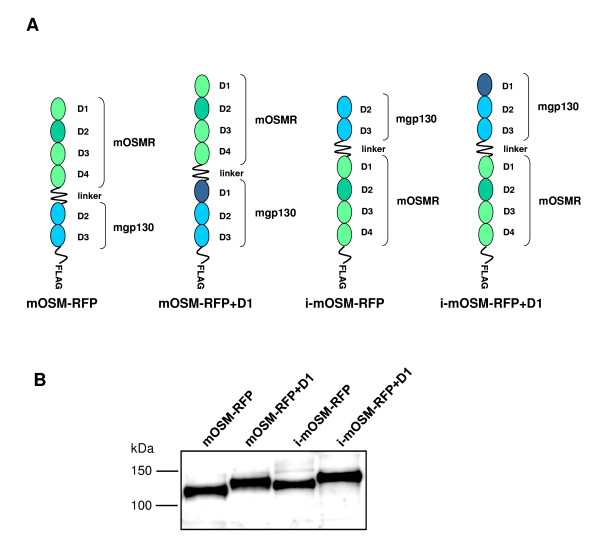
**Construction and expression of mOSM-RFPs**. (A) Schematic representation of the four OSM-RFPs analyzed in this study. (B) Supernatants of HEK293 cells were collected 48 h after transfection with expression vectors encoding the indicated mOSM-RFPs. 10-fold concentrated supernatants were analyzed by SDS-PAGE and Western blotting using a FLAG antibody.

We generated four different murine oncostatin M receptor fusion proteins (mOSM-RFPs) (Figure 1A). The first protein (mOSM-RFP) is built up in analogy to the recently published receptor fusion protein for the inhibition of murine LIF (mLIF-RFP) [6]. It consists of the four N-terminal domains of the murine OSM receptor (mOSMR) and domains D2 and D3 of murine gp130 (mgp130) connected by a flexible polypeptide linker. We [17] and others [18] have shown that the N-terminal domain D1 of gp130 is dispensable for signal transduction in response to OSM. Another report suggests a functional role of D1 of gp130 in OSM-binding [19]. Moreover, we have shown that the addition of a single domain, even if not involved in ligand-binding, can strongly enhance the expression of a receptor fusion protein [7]. Therefore, we decided to construct another fusion protein that includes D1 of mgp130 (mOSM-RFP+D1, Figure 1A). To assess the importance of the order of the receptor fragments we also constructed inverted receptor fusion proteins with the mgp130 fragment preceding the mOSMR fragment (i-mOSM-RFP and i-mOSM-RFP+D1, Figure 1A).

Human embryonic kidney (HEK293) cells were transfected with expression vectors encoding the four mOSM-RFPs. Supernatants of the cells were concentrated 10-fold and analyzed by Western blotting using an antibody directed against the FLAG-tag of the mOSM-RFPs. All four proteins were expressed (Figure 1B) indicating that neither the presence of D1 of gp130 nor the order of the receptor fragments has a major influence on protein expression. However, the yield of i-mOSM-RFP was always somewhat lower compared to the other fusion proteins. The apparent molecular masses are in the range of 120 to 140 kDa which is in agreement with the expected molecular masses of the glycosylated mOSM-RFPs.

### 2. Inhibitory activity and specificity of mOSM-RFPs

**Figure 2:**
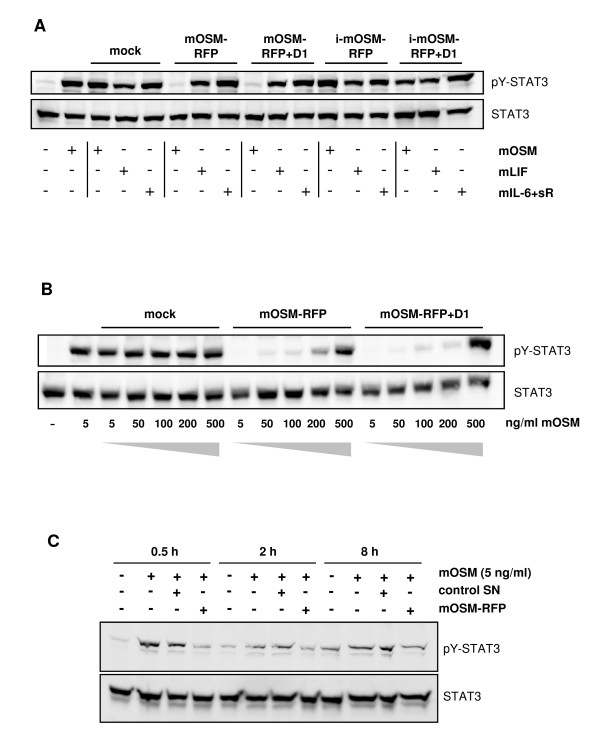
**Inhibitory activities and specificity of mOSM-RFPs**. (A) MEF were stimulated for 20 min with mOSM (5 ng/ml), mLIF (5 ng/ml) or mIL-6 (20 ng/ml) and murine soluble IL-6 receptor (sR, 500 ng/ml) in the presence of 5% of 10-fold concentrated supernatants of HEK293 cells expressing the respective mOSM-RFPs (resulting in a 6-fold molar surplus of mOSM-RFPs over mOSM) or of mock-transfected cells as indicated. (B) MEF were stimulated for 20 min with varying amounts of mOSM in the presence of 5% of 10-fold concentrated supernatants of HEK293 cells expressing mOSM-RFP or mOSM-RFP+D1 or of mock-transfected cells as indicated. Cellular lysates were analyzed for tyrosine phosphorylation of STAT3 and total STAT3 by Western blotting. (C) Fao hepatoma cells were stimulated for the indicated times with mOSM (5 ng/ml) in the presence of 2% of 10-fold concentrated supernatants of HEK293 cells expressing mOSM-RFP or corresponding supernatant of mock-transfected cells as indicated. Cellular lysates were analyzed for tyrosine phosphorylation of STAT3 and total STAT3 by Western blotting.

To analyze the activities of the mOSM-RFPs, murine embryonic fibroblasts (MEF) were stimulated with mOSM, mLIF and mIL-6 plus murine soluble IL-6 receptor (sR) in the presence of concentrated supernatants of HEK293 cells transfected with the expression vectors encoding the mOSM-RFPs or mock vector. Lysates of the MEF were analyzed for tyrosine phosphorylation of STAT3 by Western blotting (Figure 2A). Both mOSM-RFP and mOSM-RFP+D1 block the activity of mOSM but not of mLIF or mIL-6/sR indicating that these receptor fusion proteins are specific inhibitors of mOSM. Most interestingly, the inverted receptor fusion proteins are devoid of any activity under the conditions used in this assay. Therefore, in the following experiment only mOSM-RFP and mOSM-RFP+D1 were used.

MEF were stimulated with increasing amounts of mOSM in the presence of concentrated supernatants of HEK293 cells transfected with the expression vectors encoding mOSM-RFP, mOSM-RFP+D1 or mock vector. Lysates of the MEF were analyzed for tyrosine phosphorylation of STAT3 by Western blotting (Figure 2B). As soon as the mOSM concentration exceeds 200 ng/ml STAT3 activation is also detectable in the presence of the mOSM-RFPs. Thus, the tested mOSM-RFPs are saturable. This speaks for mOSM-binding in solution rather than blocking of the membrane-bound cellular receptors by a complex of mOSM and OSM-RFPs. This experiment also shows that mOSM-RFP and mOSM-RFP+D1 have comparable inhibitory activities. No increase in the STAT3 phosphorylation with increasing mOSM concentrations is seen in the mock lanes because at 5 ng/ml mOSM the cellular response is already saturated.

To test the inhibitory activity of mOSM-RFP on sustained STAT3 activation Fao rat hepatoma cells were stimulated with mOSM for prolonged periods of times (Figure 2C). Even after 8 h of stimulation mOSM-RFP reduces STAT3 activation to background levels.

### 3. mOSM-RFP blocks OSM-induced nuclear accumulation of STAT3 and SOCS3 gene induction

**Figure 3:**
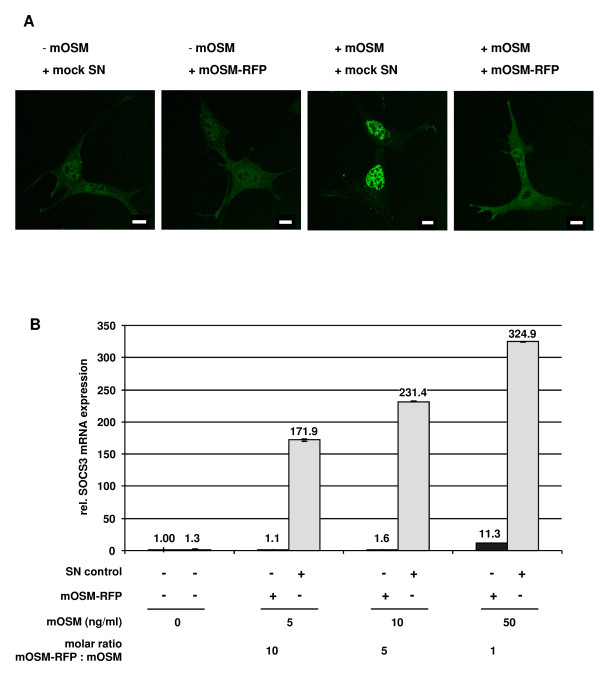
**Effect of mOSM-RFP on mOSM-induced nuclear translocation of STAT3, STAT3-mediated gene induction**. (A) MEF^ΔSTAT3 ^stably transfected with STAT3-eGFP were stimulated with 5 ng/ml mOSM for 20 min in the presence of 5% of 10-fold concentrated supernatant containing mOSM-RFP or supernatant from mock transfected cells (mock SN) as indicated. Cells were fixed and analyzed by confocal microscopy. Scale bars represent 10 μm. (B) MEF were stimulated for 45 min with varying amounts of mOSM in the presence of mOSM-RFP as indicated. Induction of mSOCS3 mRNA was quantified by qrt-PCR. mHPRT mRNA served as an internal standard.

Two further experiments were performed to analyze whether STAT3 responses downstream of tyrosine phosphorylation namely STAT3 nuclear accumulation and STAT3-mediated gene induction are blocked by mOSM-RFP. MEF lacking endogenous STAT3 (MEF^ΔSTAT3^) were stably transfected with STAT3-eGFP. These cells show the typical distribution of latent STAT3 which is found in the cytoplasm as well as in the nucleus [20] (Figure 3A). Upon addition of mOSM-RFP the distribution of STAT3-eGFP does not change. Stimulation with mOSM leads to rapid nuclear accumulation of STAT3-eGFP. Nuclear accumulation of STAT3-eGFP is blocked in the presence of mOSM-RFP.

The feedback inhibitor SOCS3 is a well established STAT3 target gene. SOCS3 gene induction in response to mOSM was measured by quantitative real-time PCR (qrt-PCR) (Figure 3B). The concentration of mOSM-RFP was estimated by Western blot titration taking advantage of the known concentration of another FLAG-tagged protein (Additional file 1). Therefore, in this and the following assays the molecular ratios of mOSM and mOSM-RFP could be properly adjusted. At a 10- and 5-fold molar excess mOSM-RFP completely inhibits mOSM mediated induction of the SOCS3 gene. Even at a molecular ratio of 1:1 most of the mOSM response is blocked by mOSM-RFP. Thus, mOSM-RFP is a specific and potent inhibitor of mOSM activity.

### 4. Comparison of mOSM-RFP with a mOSM neutralizing antibody

**Figure 4:**
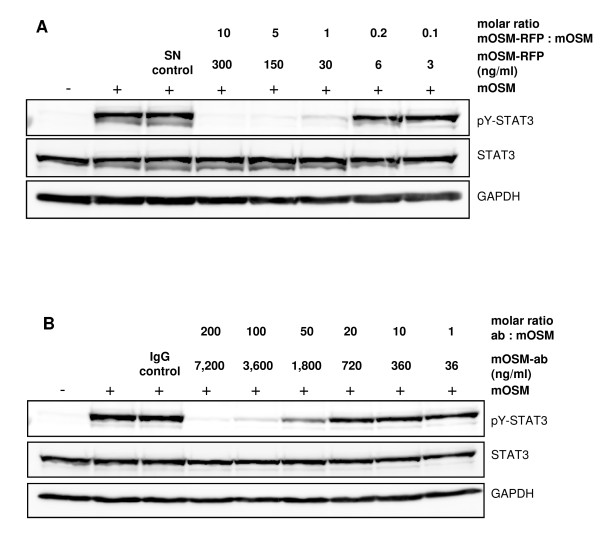
**Comparison of the activities of mOSM-RFP and a mOSM-neutralizing antibody**. MEF were stimulated for 20 min with 5 ng/ml mOSM in the presence of (A) mOSM-RFP or (B) a neutralizing polyclonal mOSM antibody at varying concentrations as indicated. Cellular lysates were analyzed for tyrosine phosphorylation of STAT3 and total STAT3 by Western blotting. GAPDH was detected as another loading control.

The inhibitory potency of mOSM-RFP was compared with a commercially available, affinity-purified neutralizing polyclonal mOSM antibody (mOSM-ab). MEF were stimulated with a constant amount of mOSM and different concentrations of mOSM-RFP (Figure 4A). In agreement with the experiment shown before (Figure 3B) a 10- to 5-fold molar excess of mOSM-RFP is sufficient to block mOSM activity. Even at a 1:1 ratio the STAT3 phosphorylation is strongly reduced. When a similar experiment is performed with mOSM-ab instead of mOSM-RFP a 50- to 100-fold molar excess of the antibody is needed to achieve a marked reduction of STAT3 phosphorylation. Thus, mOSM-RFP is a much more potent mOSM inhibitor than an affinity-purified polyclonal antibody.

### 5. Analysis of mOSM/mOSM-RFP complexes by blue-native PAGE

**Figure 5:**
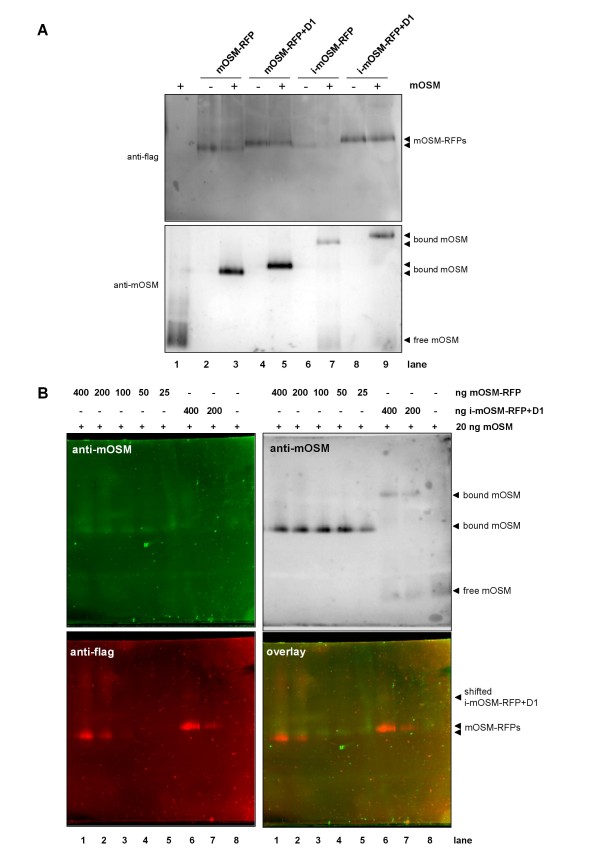
**Complexes of mOSM-RFPs and mOSM analyzed by bn-PAGE**. (A) 35 ng of mOSM were incubated with 200 ng of the respective mOSM-RFP (with the exception of i-mOSM-RFP that is less efficiently expressed and therefore 200 ng could not be achieved) in 50 μl for 30 min. Subsequently Coomassie Brilliant blue G-250 was added and the protein complexes were separated on a native gradient gel (4-16% PAA). After blotting of the proteins to a PVDF membrane mOSM-RFPs were detected using a FLAG antibody (upper panel). After stripping of the blot mOSM was detected using a mOSM antibody (middle panel). (B) bn-PAGE was performed as described in (A) with the protein amounts indicated in the figure. After blotting, detection of the proteins was performed with primary goat-anti-mOSM and mouse-anti-flag antibodies followed by secondary rabbit-anti-goat-Cy2 and donkey-anti-mouse-Cy3 antibodies. Fluorescence was detected with a fluorescence scanner. Afterwards the mOSM antibody was visualized by ECL using a matching HRP-conjugated secondary antibody.

Complexes formed between mOSM and mOSM-RFPs were analyzed by blue-native PAGE (bn-PAGE). For this purpose mOSM was preincubated with the four different mOSM-RFPs in the presence of an excess of bovine serum albumin (120 μg/ml). Proteins and protein complexes were separated by bn-PAGE and analyzed by Western blotting with a FLAG antibody for the detection of the OSM-RFPs (Figure 5A, upper panel) and after stripping of the blot with a mOSM antibody (Figure 5A, lower panel). Upon addition of mOSM no shifted bands of mOSM-RFP and mOSM-RFP+D1 are visible. However, the bands are weaker in the lanes where mOSM has been added (Figure 5A, upper panel, compare lanes 2, 3 and 4,5).

Detection of mOSM (Figure 5A, lower panel) reveals that in the presence of mOSM-RFP or mOSM-RFP+D1 the band of mOSM is completely shifted to approximately the height of the receptor fusion proteins. mOSM is only partially shifted by i-mOSM-RFP and i-mOSM-RFP+D1 but to a much higher apparent molecular mass compared to the free i-mOSM-RFPs. The receptor fusion proteins are not detectable in the shifted bands. Quantitative analysis of the blots revealed that i-mOSM-RFP and i-mOSM-RFP+D1 have a considerably lower capacity in shifting mOSM than the mOSM-RFPs (data not shown).

**Figure 6:**
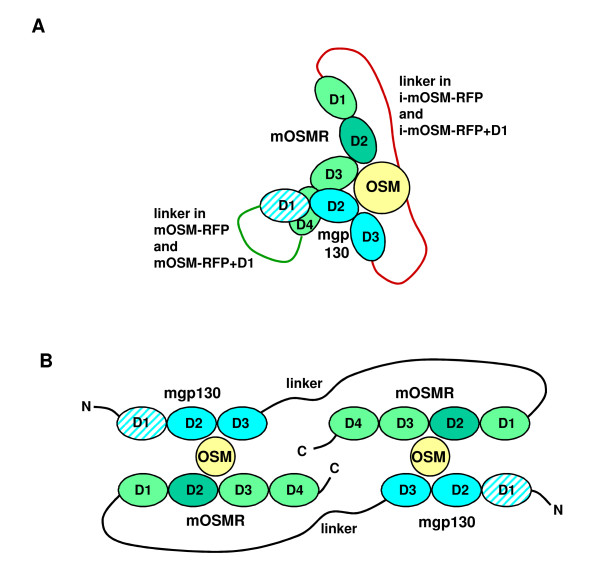
**Proposed modes of action of mOSM-RFPs**. (A) Arrangement of the domains of mOSMR and mgp130 in analogy to the closely related LIF receptor complex [22]. The green line depicts the linker in the functional inhibitors mOSM-RFP and mOSM-RFP+D1, the red line depicts the required linker for functional inverted mOSM-RFPs. (B) Schematic representation of a heterotetrameric complex of i-mOSM-RFP or i-mOSM-RFP+D1 with mOSM. Note that in three dimensions the heterotetrameric complex can be arranged in a way that the linkers are much shorter.

To clarify the identities of the bands another bn-PAGE was performed with different concentrations of mOSM-RFP and i-mOSM-RFP+D1. After blotting of the gel the FLAG-tags of the fusion proteins and mOSM were detected using antibodies with different fluorescent labels. The blots were analyzed using a fluorescence scanner (Figure 5B). mOSM shifted by mOSM-RFP is visible (Figure 5B, upper left image) although the fluorescence detection is not as sensitive as the enhanced chemiluminescence detection (Figure 5B, upper right image) where the mOSM shifted by i-mOSM-RFP+D1 and the non-shifted mOSM are also visible. The fluorescence detection of the FLAG-tag (Figure 5B, lower left image) and the overlay of the two fluorescence detections (Figure 5B, lower right image) reveal that the shifted mOSM is of lower electrophoretic mobility than mOSM-RFP. Apparently, the FLAG-tag of mOSM-RFP is nearly completely masked upon complex formation with mOSM so that only very faint bands are visible (e.g. Figure 5B, lower panels, lane 6). From the electrophoretic mobilities and the shift of the bands we conclude that mOSM is bound by mOSM-RFP or mOSM-RFP+D1 in a 1:1 complex (Figure 6A). We propose that mOSM binds to dimers of the i-mOSM-RFPs (Figure 6B). A propensity for dimer formation of the i-mOSM-RFPs is evident from SDS-PAGE where the dimers can be detected even under denaturing conditions (Additional file 2).

## Discussion

We developed novel receptor fusion proteins for the inhibition of murine OSM. The receptor fusion protein mOSM-RFP consisting of domains D1-D4 of mOSMR connected by a flexible peptide linker to domains D2-D3 of mgp130 is a potent inhibitor of mOSM with respect to STAT3 phosphorylation (Figure 2), STAT3 nuclear translocation and STAT3-mediated gene induction (Figure 3). Addition of D1 of mgp130 does neither improve protein expression (Figure 1) nor inhibitory activity (Figure 2B) confirming that domains D2-D3 of gp130 are sufficient for complex formation with OSM [17,21]. Our fusion proteins for the inhibition of murine OSM differ from a previously described fusion protein for the inhibition of human OSM that consisted of the first 428 amino acids of soluble hOSMR fused to 51 amino acids (aa 767-817) of hgp130 [3]. A more recently published receptor fusion protein for the inhibition of human IL-31 consisting of D1-D4 of hOSMR and D1-D2 of hIL-31R [4] is built up in analogy to our formerly described mLIF-RFP [6] and therefore resembles mOSM-RFP described in this study. Previously described cytokine traps consist of soluble receptors fused to Fc-fragments. These cytokine traps require the expression of two genes. After protein expression, homodimers must be separated from the desired heterodimeric proteins [5].

Compared to mOSM-RFP and mOSM-RFP+D1 the inverted proteins i-mOSM-RFP and i-mOSM-RFP+D1 are weak OSM-binding proteins (Figure 5) and therefore unsuited as inhibitors (Figure 2A). This initially puzzling observation might be explained at least in part by the analysis of OSM/OSM-RFP complexes with bn-PAGE (Figure 5). mOSM-RFP and mOSM-RFP+D1 when bound to mOSM form a 1:1 complex that is in agreement with the proposed stoichiometry of the OSM receptor complex consisting of one molecule of each OSM, OSMR and gp130 [22]. When the domains of OSMR and gp130 are arranged in a way as proposed for the closely related LIF receptor complex ([22], Figure 6A) it is evident that the C-terminus of domain D4 of OSMR is much closer to the N-terminus of gp130 than the C-terminus of gp130 to the N-terminus of OSMR. The latter distance is relevant for the inverted mOSM-RFPs and might be too long to be bridged by the 43 amino acid linker used in our fusion proteins. Therefore, the complex adopts an alternative conformation of 2:2 stoichiometry as shown in Figure 6B. Apparently, this alternative complex is of lower affinity than the native receptor complex.

Gp130 is the common receptor subunit of the IL-6 family of cytokines comprising IL-6, OSM, LIF, IL-11, IL-27, ciliary neurotrophic factor, cardiotrophin-1, cardiotrophin-like cytokine and neuropoetin [23]. None of these cytokines binds with high affinity to gp130 alone. For high affinity-binding a second more specific receptor subunit is required [24]. Accordingly, mOSM-RFP is a mOSM-specific inhibitor that does not inhibit the related cytokines mIL-6 and mLIF (Figure 2A) that require mIL-6R and mLIFR, respectively, for high affinity-binding. Conversely, the mIL-6 inhibitor mIL-6-RFP and the mLIF inhibitor mLIF-RFP do not inhibit mOSM [6,7]. Thus, shared cytokine receptors are useful for the design of specific cytokine inhibitors. For this reason, the receptor fusion protein approach can be applied to the remaining cytokines of the IL-6 family but also to cytokines that use other shared receptors such as the IL-2 family and the IL-3 family of cytokines using the common γ-chain and the common β-chain, respectively. mOSM-RFP will be a useful tool to decipher the controversially discussed role of mOSM in murine models of inflammatory diseases [8].

## Conclusions

The functional OSM-RFPs presented in this study and the previously described RFPs are highly specific and potent cytokine inhibitors. RFPs are encoded by a single gene. This offers an advantage with respect to gene delivery approaches compared to cytokine traps based on soluble receptors fused to Fc-tags [5] or antibodies which are composed of two different protein chains. RFPs can be conveniently expressed as single gene constructs by viral gene transfer or in transgenic animals. Thus, RFPs offer numerous possibilities to test the effect of cytokine inhibition in immune responses, in animal models of human diseases and finally in gene therapy.

## Methods

### Recombinant plasmids

The expression vectors pcDNA5/FRT/TO-mOSM-RFP, pcDNA5/FRT/TO-mOSM-RFP+D1, pcDNA5/FRT/TO-i-mOSM-RFP and pcDNA5/FRT/TO-i-mOSM-RFP+D1 were constructed using previously described pSVL-mLIF-RFP [6] and pcDNA3.1-mIL6-RFP [7] expression vectors and the pcDNA5/FRT/TO vector of the Flp-In system (Invitrogen, USA). The mOSMR fragment was amplified by RT-PCR from total MEF mRNA.

All expression constructs of the mOSM-RFPs are built up similarly on the backbone of the pcDNA5/FRT/TO vector. A Kozak consensus sequence (GCC ACC) is followed by the leader sequence of preprotrypsin for optimal mRNA translation and protein secretion, respectively. The receptor fragments are joined by a polypeptide linker of 43 amino acids (PGGSAAATRG SAGSGGSATG SGSAAGSGDS VAAGSGGGSG SAS). OSM-RFP and OSM-RFP+D1 contain a C-terminal FLAG-tag (DYKDDDDK), the inverted OSM-RFPs contain an additional hexa-histidine-tag. The mOSMR-fragment is identical in all four mOSM-RFPs and covers Glu24-Pro427 corresponding to domains D1-D4. Domains D2-D3 and domains D1-D3 of mgp130 encompass Ser122-Pro324 and Gln23-Pro324, respectively.

### Cytokines, cytokine receptors and antibodies

Murine OSM, murine IL-6 and murine soluble IL-6Rα (sR) were purchased from R&D Systems (MN, USA), murine LIF (ESGRO^®^) from Chemicon (CA, USA). The neutralizing goat anti-mOSM antibody was purchased from R&D Systems (MN, USA), an IgG control antibody from Immunotools (Friesoythe, Germany).

### Cell culture and transfection

Murine embryonic fibroblasts (MEF) and human embryonic kidney cells (HEK293) were cultivated in Dulbecco's Modified Eagle Medium (DMEM) with GlutaMax™ (Invitrogen, CA, USA) supplemented with 10% FCS, 100 U/ml penicillin and 100 μg/ml streptomycin (BIO-Whittaker, Verviers, Belgium). For the cultivation of Fao rat hepatoma cells DMEM/F12 was used. The cells were incubated at 37°C in a water-saturated atmosphere at 5% CO_2_. HEK293 cells were transfected with TransIT^® ^LT-1 transfection reagent (Mirus, WI, USA) in a ratio of 1 μg DNA to 3 μl transfection reagent. Medium was exchanged to DMEM with GlutaMax™ without FCS after 4 hours. Supernatants were harvested after 48 h, cleared by centrifugation and 10-fold concentrated in Vivaspin 20 centrifugal concentrators (Sartorius, Göttingen, Germany).

### Quantification of mOSM-RFP by Western blotting

FLAG-tagged OSM-RFP was quantified by calibration with known amounts of FLAG-tagged hIL-6-RFP by Western blotting using a FLAG antibody (SIGMA-Aldrich, Taufkirchen, Germany). The intensity of immunodetected bands was quantified using the Fujifilm MultiGauge software.

### Preparation of cell lysates, SDS-PAGE and immunoblotting

MEF or Fao cells were grown on 6-well plates to 70% confluence. A mixture of cytokine and mOSM-RFP, neutralizing mOSM antibody or control supernatant, respectively, was preincubated in 1 ml DMEM for 30 min and afterwards added to the cells for 20 min. Subsequently, cells were lysed with RIPA lysis buffer (50 mM Tris-HCl, pH 7.4, 150 mM NaCl, 1 mM EDTA, 0.5% Nonidet P-40, 1 mM NaF, 15% glycerol, 20 mM β-glycerophosphate, 1 mM Na_3_VO_4_, 0.25 mM phenylmethylsulfonylfluoride, 5 μg/ml aprotinin, and 1 μg/ml leupeptin). The lysates were analyzed by Western blotting using antibodies directed against phosphotyrosine705-STAT3 (Cell Signaling Technology, MA, USA), STAT3 (BD Transduction Laboratories, CA, USA), GAPDH (Santa Cruz, CA, USA) and HRP conjugated secondary antibodies (Dako, Hamburg, Germany). All first antibodies were used in a 1:1.000 dilution, all secondary antibodies in a 1:2.000 dilution in TBS-N (20 mM Tris-HCl, pH 7.6, 137 mM NaCl and 0.1% Nonidet P-40). Bound antibodies were detected by chemiluminescence (ECL, Millipore, MA, USA). Membranes were stripped in 62.5 mM Tris-HCl, pH 7.6 containing 2% SDS and 0.08% β-mercaptoethanol for 25 min at 70°C before a second detection was performed.

### Blue-native PAGE

mOSM and concentrated supernatants with the respective mOSM-RFP were preincubated for 30 min at RT in the presence of 120 μg/ml bovine serum albumin to allow formation of protein complexes which were subsequently separated on native 4-16% bis-tris gels in running buffer containing Coomassie Brilliant Blue G-250. Gels were analyzed by Western blotting using antibodies directed against mOSM (goat-anti-mOSM, R&D Systems, MN, USA), FLAG (mouse-anti-flag, SIGMA-Aldrich, Taufkirchen, Germany) and HRP conjugated secondary antibodies for ECL detection or rabbit-anti-goat-Cy2 and donkey-anti-mouse-Cy3 for fluorescence detection (Dako, Hamburg, Germany). Fluorescence was detected with a fluorescence scanner (Typhoon, Amersham). Novex bis-tris gels (4-16%), loading and running buffers were purchased from Invitrogen™ (NativePAGE™, CA, USA).

### Confocal fluorescence microscopy and live cell imaging

The confocal imaging was performed with a Zeiss LSM 510Meta confocal microscope (Zeiss, Jena, Germany). eGFP fluorescence was detected using the 488 nm line of the argon laser, a 488 nm dichroic mirror and a 500-530 nm bandpass filter. MEF^ΔSTAT3 ^cells stably transfected with STAT3-eGFP were cultured on glass coverslips for 48 h. mOSM and concentrated supernatant containing mOSM-RFP were preincubated in 1 ml DMEM for 30 min at RT. Cells were stimulated for 20 min or left untreated. For fixation the cells were incubated in 3.7% paraformaldehyde for 15 min and washed twice with PBS containing 1 mM MgCl_2 _and 0.1 mM CaCl_2 _(PBS^++^). Afterwards the cells were quenched with 50 mM NH_4_Cl in PBS^++ ^for 5 min, dipped in water and mounted with ImmuMount (Shandon, PA, USA).

### Quantitative real-time PCR

MEF were grown on 6-well plates to 70% confluence. After preincubation of mOSM and mOSM-RFP in 1 ml DMEM for 30 min MEF were stimulated for 45 min and subsequently lysed. RNA was extracted using the RNeasy Kit (Qiagen, Hilden, Germany). After reverse transcription using Omniscript-RT-PCR-Kit (Qiagen, Hilden, Germany) cDNA was amplified in duplicates in a Rotor-Gene Q (Qiagen, Hilden, Germany) using specific primers for mSOCS3 (Mm00545913_s1) and mHPRT (Mm015453399_m1) as internal standard (TaqMan^® ^Gene Expression Assay, Applied Biosystems, CA, USA). PCR reaction was carried out in a 20 μl reaction volume in the presence of 25 ng cDNA and 1 μl of primer mixture. Thermal cycling was initiated with 2 min incubation at 50°C and 10 min at 95°C followed by 40 cycles of 95°C for 15 s and 60°C for 60 s. The ΔCt method was used for quantification.

## List of abbreviations

BN: blue-native; D: domain; eGFP: enhanced green fluorescent protein; H: human or hour; HEK: human embryonic kidney; IL-6: interleukin-6; LIF: leukemia inhibitory factor; M: murine; OSM: oncostatin M; MEF: murine embryonic fibroblasts; RFP: receptor fusion protein; sR: soluble receptor; STAT: signal transducer and activator of transcription;

## Authors' contributions

LB designed and performed most of the experiments including cloning of the constructs and participtated in writing of the manuscript, AK designed plasmid constructs and contributed to most of the experiments, SK established expression of the mOSM-RFPs and performed a first characterization of the recombinant proteins, MV participated in bn-PAGE and generated and validated the stable cell line used for confocal microscopy, GMN initiated and supervised the study and wrote the manuscript. All authors read and approved the final manuscript.

## Supplementary Material

Additional file 1 **Quantification of mOSM-RFP**. Click here for file

Additional file 2 **i-mOSM-RFPs form dimers**. Click here for file
