# Supplementary material for: A receptor fusion protein for the inhibition of murine oncostatin M
Source: BMC Biotechnol. 2011 Jan 11;11:3. doi: 10.1186/1472-6750-11-3 (PMC3040522; doi:10.1186/1472-6750-11-3)
Supplement: Additional file 2 — i-mOSM-RFPs form dimers. [file 1472-6750-11-3-S2.PDF]

i-mOSM-RFPs form dimers. Different amounts of 10-fold concentrated supernatants of HEK293 cells expressing the indicated mOSM-RFPs were loaded on a SDS-PAA gel. After blotting the mOSM-RFPs were detected with a FLAG-antibody. Dimers of i-mOSM-RFPs are visible most notably at higher concentrations.

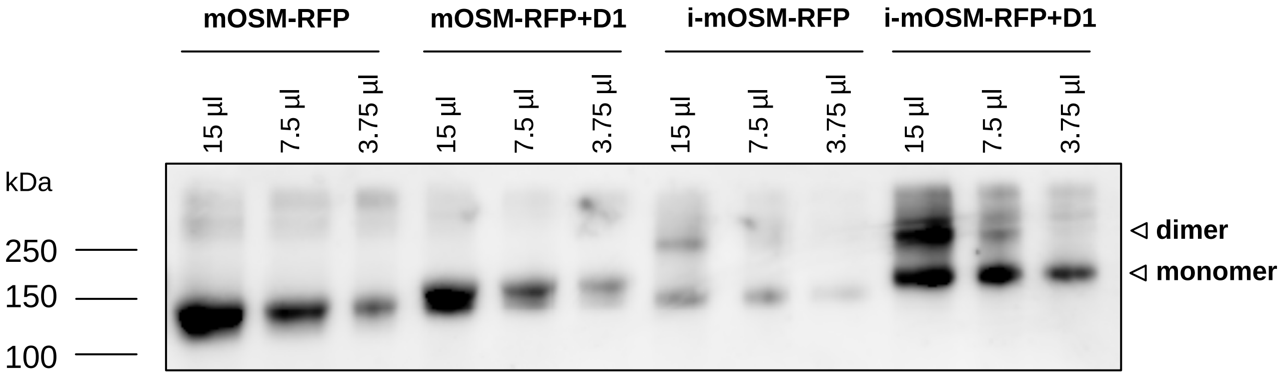

Brolund et al.
Suppl. Fig. S2
